# Enhancement of the Excitation Efficiency of the Non-Contact Magnetostrictive Sensor for Pipe Inspection by Adjusting the Alternating Magnetic Field Axial Length

**DOI:** 10.3390/s140101544

**Published:** 2014-01-16

**Authors:** Pengfei Sun, Xinjun Wu, Jiang Xu, Jian Li

**Affiliations:** School of Mechanical Science & Engineering, Huazhong University of Science and Technology, Wuhan 430074, China; E-Mails: hustpfs@mail.hust.edu.cn (P.S.); jiangxu@mail.hust.edu.cn (J.X.); smartlijian@mail.hust.edu.cn (J.L.)

**Keywords:** guided wave, magnetostriction, transmitter, excitation efficiency, alternating magnetic field

## Abstract

The non-contact magnetostrictive sensor (MsS) has been widely used in the guided wave testing of pipes, cables, and so on. However, it has a disadvantage of low excitation efficiency. A new method for enhancing the excitation efficiency of the non-contact MsS for pipe inspection using guided waves, by adjusting the axial length of the excitation magnetic field, is proposed. A special transmitter structure, in which two copper rings are added beside the transmitter coil, is used to adjust the axial length at the expense of weakening the excitation magnetic field. An equivalent vibration model is presented to analyze the influence of the axial length variation. The final result is investigated by experiments. Results show that the excitation efficiency of the non-contact MsS is enhanced in the whole inspection frequency range of the L(0,2) mode if the axial length is adjusted to a certain value. Moreover that certain axial length is the same for pipes of different sizes but made of the same material.

## 1. Introduction

**Figure 1.**
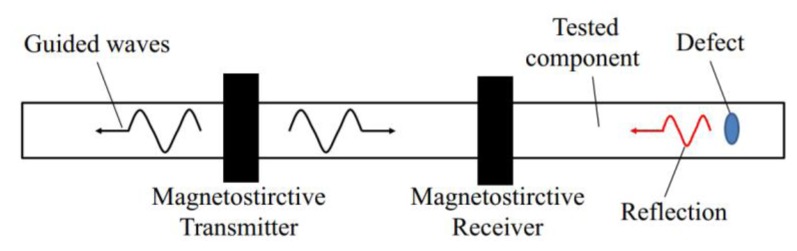
The principle of GWT using MsSs.

Magnetostrictive sensors (MsSs) have been widely used in many fields [1], such as torque sensors, position sensors, stress sensors, *etc*. MsSs also play an important role in guided wave testing (GWT), one of latest methods in the field of non-destructive testing (NDT) of pipes, rods, plates, cables and so on. In GWT, usually two MsSs, a transmitter and a receiver, work at the same time, as shown in Figure 1. The magnetostrictive transmitter produces magnetostrictive strain to generate special mechanical stress waves—guided waves—which propagate along the tested component. Guided waves will be reflected if there is any anomalous structure in the tested component, for example a crack, a through-hole, a pitting corrosion or a weld. When the reflection passes through the magnetostrictive receiver, the changes of the stress and the strain caused by the reflection can be received and transformed into an electrical signal based on the inverse magnetostrictive effect. The round trip time-of-fight (TOF) of the defect reflection can be measured and the velocity of the longitudinal guided wave can be calculated. Therefore, the location of the defect can be obtained. What's more, the defect size can be evaluated through the reflection coefficient which is defined as the ratio between the amplitude of the defect signal by the amplitude of the incident signal [2,3]. In this way, defects in the component can be inspected by GWT using MsSs.

**Figure 2.**
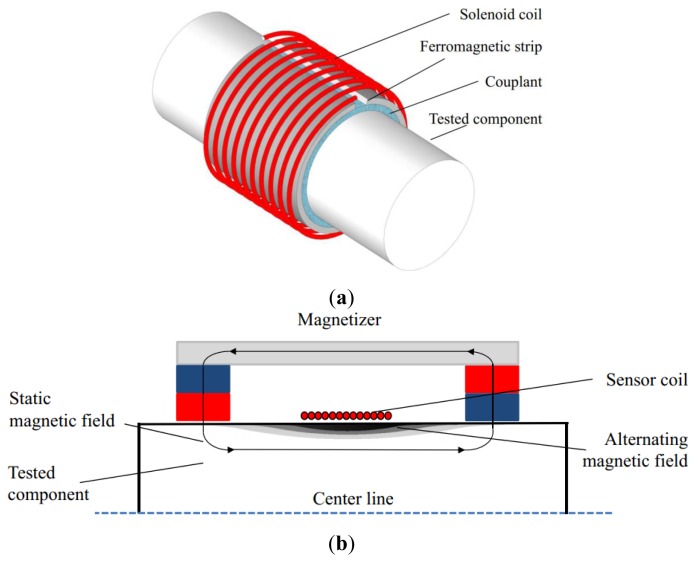
The structures of MsSs used in GWT. (**a**) The contact MsS. (**b**) The non-contact MsS.

Two types of MsSs have been used in GWT, the contact sensor and non-contact sensor. Figure 2a shows the structure of a contact sensor [4,5], which consists of a pre-magnetized ferromagnetic strip attached at the surface of the tested component and a sensor coil. The contact sensor works based on the magnetostrictive effect of the strip itself, which is made of a material having a large magnetostriction, for example, an iron-cobalt alloy. Therefore, its performance is entirely determined by the intrinsic characteristics of the magnetostrictive material and has no relation to the tested component. New magnetostrictive materials and configurations have been developed to enhance the detectability [6,7]. Figure 2b shows the structure of a non-contact sensor, which consists of a magnetizer, using permanent magnets or a direct current (DC) coil, and a sensor coil. The non-contact sensor produces a strain directly in the detected ferromagnetic component using the magnetostrictive effect of the object itself [8]. The whole process does not need any physical contact or couplant, which means that the non-contact sensor can generate and receive the guided wave in the tested component with a gap (more than several centimeters) between the sensor and the surface of the object.

Hence, the non-contact magnetostrictive sensor (MsS) is suitable for testing of steel pipes or cables with coatings, which are usually costly or unable to be removed. Nevertheless, due to the low magnetostriction of the tested component itself, the non-contact MsS has a lower conversion efficiency and lower sensitivity compared with contact sensors. Notice that the sensitivity of the MsS is defined as a measure of the smallest defect signal which can be discernible on the inspection signal [9,10]. What's more, the magnetostriction of the tested component cannot be changed. It means that the sensitivity of the non-contact MsS, unlike the sensitivities of other MsSs, cannot be enhanced by developing new materials with higher magnetostriction.

In the literature, many other ways to obtain higher excitation efficiency for the non-contact MsS used in GWT have been tried. For a given ferromagnetic object under inspection, the strength of the static magnetic field in the component, produced by the magnetizer, determines the energy conversion efficiency from the alternating magnetic field induced by the sensor coil to the elastic field transmitting in the tested component [11,12]. A suitable static magnetic field strength will maximize the energy conversion efficiency and enhance the sensor sensitivity [13]. Moreover, the static magnetic field should be as uniform as possible to reduce the noise of the inspection signal [14]. Enhancing the excitation magnetic field by increasing the alternating current (AC) loaded in the transmitter coil is another way to improve the sensor sensitivity [13]. However, to a certain extent, these methods increase the cost and complexity of the inspection system.

Compared with the above methods, an easier way to increase sensor excitation efficiency is to develop a new sensor coil structure. In recent years, a three part coil has been developed to generate the guided wave under a specific frequency [15–17]. Once the inspection frequency is changed, the excitation efficiency of the three part coil will decrease distinctly. However, the excitation efficiency of GWT increases as the inspection frequency becomes higher, but the inspection range narrows at the same time. Therefore, GWT is usually done under different inspection frequencies to combine the requirements of excitation efficiency and inspection range, and a single inspection frequency cannot meet these requirements. A method for improving the excitation efficiency of the magnetostrictive sensor used in GWT under a wider inspection frequency range is needed.

In this paper, we present a method to enhance the excitation efficiency of the magnetostrictive transmitter for pipe inspections by optimizing the axial length of the excitation magnetic field. What's more, the enhancement can be realized under the frequency range of L(0,2) mode rather than a single frequency.

To achieve this objective, a special structure, adding two copper rings beside the transmitter coil, is used here to adjust the axial length. The structure is similar to the one used by Seco *et al.* [18], however, the focus of this paper is different from the previous paper, in which the authors focused on compensating the hysteresis occurred in the magnetostrictive linear position sensor. The details of the structure to adjust the axial length of the excitation magnetic field are presented in Section 2. In Section 3, the influence of the axial length of the excitation magnetic field on the excitation efficiency is analyzed theoretically. Section 4 validates the theoretical analysis by experiments. Finally, a brief conclusion is given in Section 5.

## 2. The Method for Adjusting the Axial Length of the Excitation Magnetic Field

### 2.1. The Axial Length of the Excitation Magnetic Field

**Figure 3.**
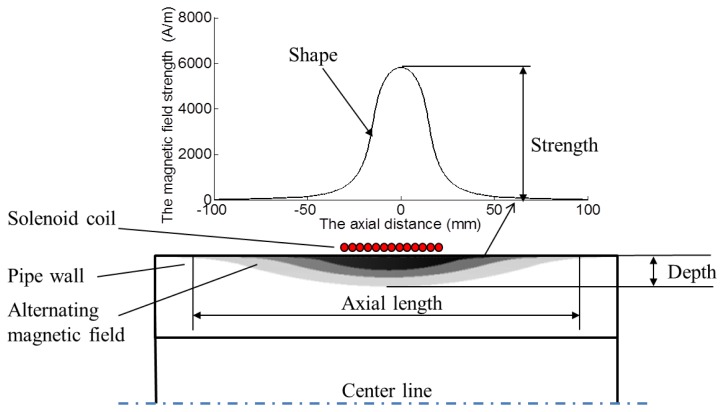
The parameters of the alternating magnetic field produced by a solenoid coil.

For an alternating magnetic field produced by the transmitter coil in a pipe, there are some parameters could be used to describe the distribution of the field, including the strength, the depth, the axial length, and the shape of the distribution, as shown in Figure 3.

The strength stands for the maximum of the magnetic field strength of the field. The depth is the skin depth and the axial length is the axial range where the alternating magnetic field exists. The shape of the distribution is the geometrical shape of the magnetic field strength in the whole skin area when the above three parameters are determined. The alteration of transmitter structures may change these four parameters, and the four parameters influence the excitation efficiency. Usually the axial length of the excitation magnetic field is influenced by the material of the pipe and the structure of the transmitter coil. However, the conventional structure cannot meet the requirement of adjusting the axial length conveniently. A special structure is needed to adjust the axial length. A structure, in which two copper rings are added beside the transmitter coil, is used in this paper. The detailed comparison between the conventional structure and the special structure is presented as follows.

### 2.2. The Conventional Structure

**Figure 4.**
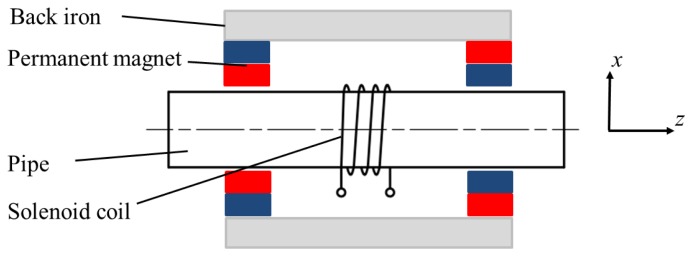
The conventional structure of the transmitter for the longitudinal guided wave testing.

The conventional structure of the non-contact magnetostrictive transmitter is shown in Figure 4. It consists of back irons, permanent magnets and a solenoid coil providing the alternating magnetic field. A static magnetic field with certain strength is produced in the pipe wall by the back irons and permanent magnets. An alternating magnetic field is applied on the pipe when an alternating current is provided to the transmitter coil. Once the coil structure changes, the parameters of the alternating magnetic field would be different. Usually there are the three structure parameters (turns, the lift-off and the width). The turns only influence the strength of the alternating magnetic when the other two are constant. The lift-off is harmful to the excitation efficiency and should be as small as possible. Therefore it is often the variation of coil width that causes changes in the axial length of the alternating magnetic field. The finite element method (FEM) based on Ansys 12.0 is used here to calculate the alternating magnetic fields produced by coils with different widths.

**Figure 5.**
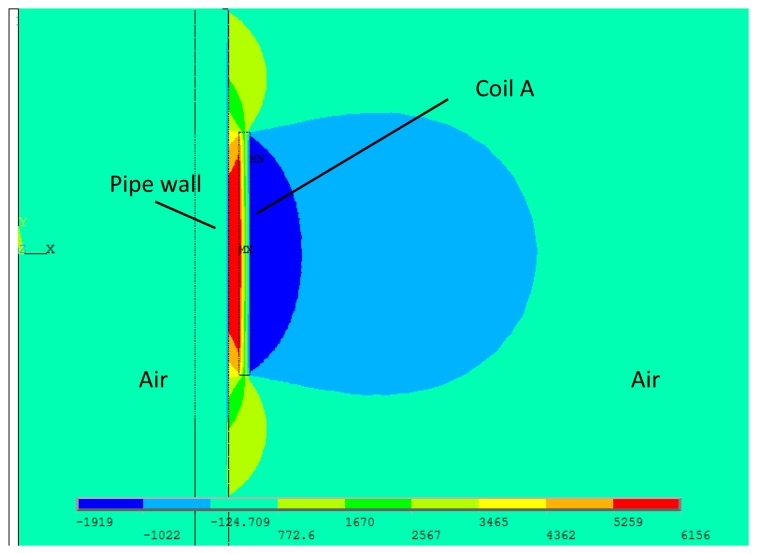
The alternating magnetic field produced by the conventional structure.

**Figure 6.**
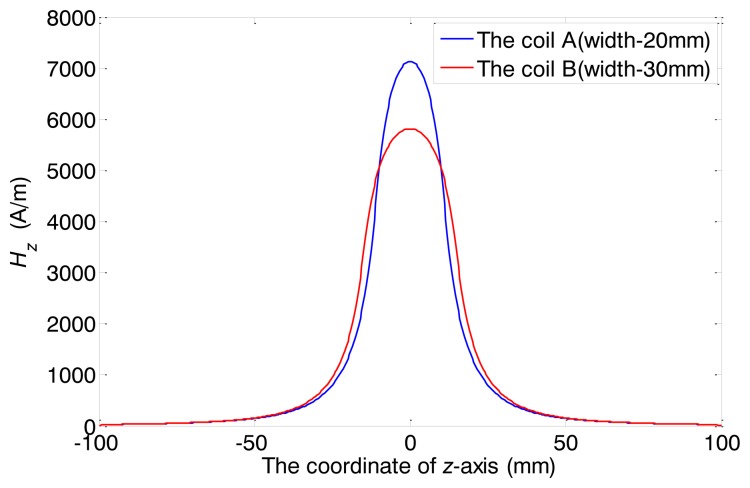
The axial magnetic field strength (*H_z_*) produced by two transmitter coils with two different widths.

In the FEM model, only the alternating magnetic field is of concern. Thus the existence of the back irons and the permanent magnets are ignored. Two different widths have been chosen, 20 mm and 30 mm. The former one is defined as the coil A, and the latter the coil B. The turn numbers of both coils are 20 and the inner diameters are 40 mm. Both coils are wrapped by copper wires of 1 mm diameter. The outer diameter and the wall thickness of the pipe are 38 mm and 3 mm, respectively. The relative magnetic permeability is 200 and the electrical conductivity is 1 × 10^7^ S/m. The direction of the pipe axis is defined as *z* axis, and the XOY plane overlaps with the middle of the model, as well as the middle of the coil. A two cycle sine burst at 120 kHz is used as an excitation signal in the following discussion. In order to keep the ampere-turns per meter and the current density constant, the peak-to-peak amplitudes of the applied currents are set as 30 A, 45 A for coil A and B, respectively. The respective axial magnetic field strengths (*H_z_*) produced by the two coils are calculated, which are mainly of concern in the longitudinal mode guided wave testing. Figure 5 shows the full plot of the alternating magnetic field produced by the conventional structure. The maximum values of the *H_z_* on the surface of the pipe are shown in Figure 6.

As we can see, firstly, the strength of alternating magnetic field produced by coil A is stronger than that of coil B. Secondly, it is hard to distinguish the difference between the axial lengths of the two alternating magnetic fields. Thirdly, the both axial alternating magnetic fields are quite different from each other in the shapes of the distribution. Compared with the axial alternating magnetic field produced by coil B, the field produced by coil A is stronger in the *z* coordinate range (from −10 mm to 10 mm) and smaller in the other locations. In other words, the shape of alternating magnetic field distribution using coil A is sharper than using coil B.

To sum up, the strength, the shape and the axial length of the alternating magnetic field are all changed with the variation of coil width. Moreover, the change of the axial length is very small. Hence, the axial length is hard to adjust with the conventional structure. The relationship between the axial length and the sensor excitation efficiency cannot be obtained using the conventional structure. A special structure is needed to adjust the axial length and minimize other changes of the excitation magnetic field.

### 2.3. The Special Structure

**Figure 7.**
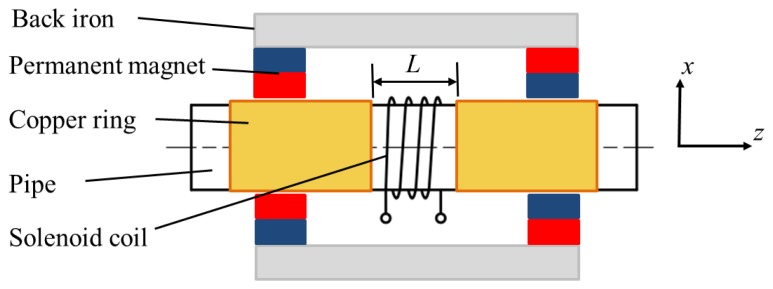
The special structure of the transmitter for the longitudinal guided wave testing.

A special structure is used in this paper as shown in Figure 7. Two rings made of shin copper sheet are placed beside the solenoid coil with an axial distance *L*. The thickness of copper ring should be larger than its skin depth. The copper rings would shield the alternating magnetic field from the pipe range below them. The axial length of alternating magnetic field is limited to the area between the two copper rings.

**Figure 8.**
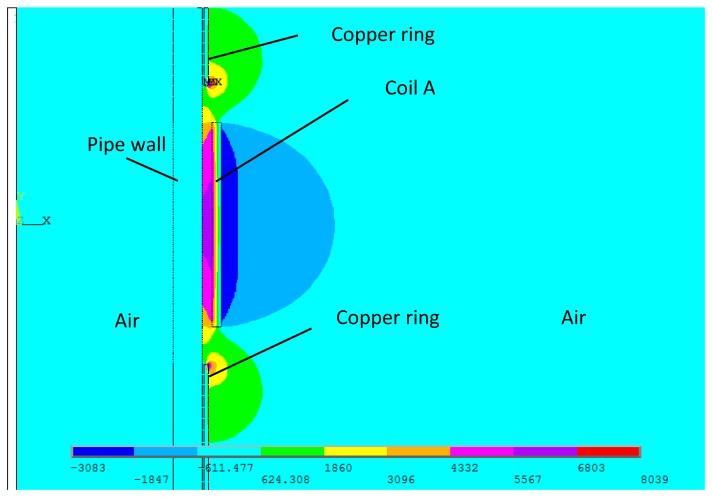
The alternating magnetic field produced by the special structure when *L* = 30 mm.

**Figure 9.**
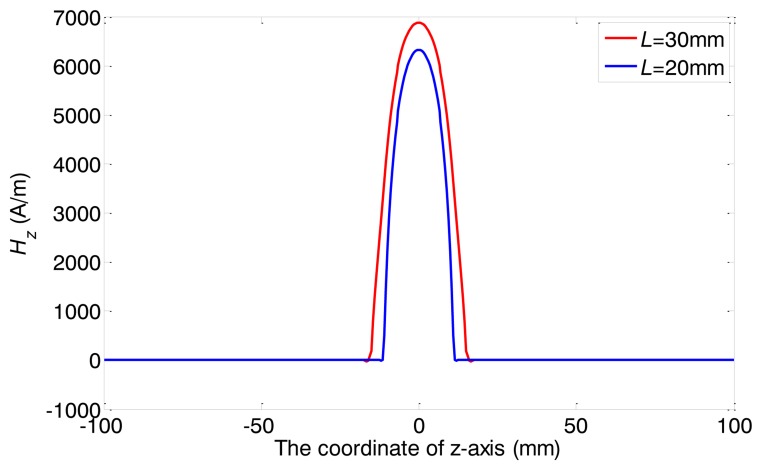
The alternating magnetic field produced by the special structure when *L* = 30 mm.

The detailed alternating magnetic field produced by this transmitter is calculated through FEM. The above coil A is here used as the transmitter coil. The excitation current flowing through the coil is a two cycle sine burst at 120 kHz. The peak to peak value is 30 A. The skin depth of the copper rings is 0.2 mm and the thickness of the copper rings should be larger than the skin depth. Hence, a thickness of 0.5 mm is chosen here. The permanent magnets are placed on them. Therefore, the static magnetic field changes little compared with the conventional structure. The parameters of pipe are the same as mentioned above. Figure 8 shows the full plot of the alternating magnetic field produced by the special structure when *L* = 30 mm. Figure 9 shows FEM calculation results of the axial alternating magnetic-field components (*H_z_*) on the surface of the pipe when the *L* is 20 and 30 mm.

Because of the existence of the two copper rings, the axial lengths of the alternating magnetic fields are both close to *L* under two different conditions. Besides, the shapes of distributions are much more similar than the conventional structure as shown in Figure 5. Therefore, the special structure can be used to study the relationship between the axial length of alternating magnetic field and the excitation efficiency.

## 3. Theory

### 3.1. The Equivalent Model

**Figure 10.**
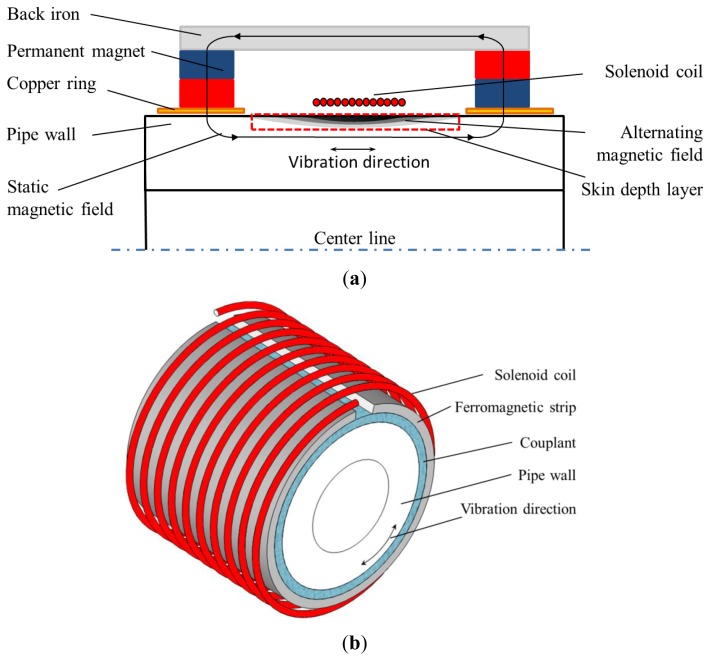
The guided wave generation progress (**a**) using the non-contact transmitter, (**b**) using the contact transmitter.

From the above analysis, the alternating magnetic field mainly just exists in the length between the two copper rings, where the static magnetic field produced by permanent magnets also exists. The main components of both fields are along the pipe axis. Based on the magnetostriction effect, an alternating magnetostrictive force is produced in the skin depth area and its direction is also mainly along the pipe axis, as shown in Figure 10a. An axial vibration is generated in the skin depth area.

An ultrasonic wave is then directly induced by the vibration. According to Snell's law, the wave undergoes the mode conversion, reflection and refraction at the inner and outer face of the pipe wall [19]. At some distance away from the transmitter, the wave will no longer be individually identifiable, but will turn into a wave packet and propagate along the pipe axis, which is called the longitudinal guided wave. It is the skin depth area that acts as a vibration source, which directly generates the guided wave.

This action is similar to the action of ferromagnetic strip when using the contact transmitter as shown in Figure 10b. The pre-magnetized ferromagnetic strip is attached at the surface of the pipe usually by the epoxy as the couplant. In the first place, the solenoid coil surrounds the strip and generates a circumferential vibration in the strip. Then the vibration is coupled into the pipe wall and ultrasonic wave is formed in the pipe. Finally, the torsional guided wave propagates along the pipe through a series of mode conversions, reflections and refractions.

According to the above analysis, both the skin depth area and the ferromagnetic strip are locations where vibration occurs firstly and could be regarded as the vibration sources during the generation progress of guided waves. When the strength of the vibration source increases, the strength of guided waves would be stronger. For the ferromagnetic strip, its vibration strength is greatly impacted by its natural frequency and the excitation current frequency. The closer the excitation current frequency is to the natural frequency of the strip, the stronger is the vibration and the higher is the excitation efficiency of the contact transmitter.

Therefore, in a similar way, for the noncontact transmitter, the skin depth area, as the vibration source, can be taken into account individually without concerning other ranges of the pipe. In this way, the skin depth area could be simplified to a clamped–clamped pipe model. The length of this model is determined by the axial length of the alternating magnetic field. If the natural frequency of the equivalent model can be calculated, the relationship between the axial length and the natural frequency could be got. Then the relationship between the axial length and the excitation efficiency could be analyzed qualitatively, so the problem becomes how to calculate the natural frequency of the equivalent model.

### 3.2. The Calculation of Natural Frequency and Analysis

According the classical vibration theory, for a pipe, the natural frequency is deduced from:

(1) $$f_{n}=\frac{1}{2\pi }\sqrt{\frac{k}{m}}$$

where *k* is the stiffness of the pipe and *m* is the quality. Considering the special condition of pipe inspection using a magnetostrictive transmitter in this paper, only the axial stiffness is of concern. The axial stiffness of clamped–clamped pipe is twice that of the free pipe [20]. We can obtain the axial stiffness as:

(2) $$k=\frac{2EA}{L_{e}}$$

and the quality *m* could be deduced from:

(3) $$m=\rho AL_{e}$$

(4) $$L_{e}=C_{e}L$$

(5) $$A=A_{sk}$$

where *E* is the Young's modulus of the pipe, *A* is cross sectional area. *ρ* is the density of the pipe. *L_e_* is the length of the equivalent model, *L* is axial length of the excitation magnetic field and *C_e_* is the equivalent coefficient between *L* and *L_e_*. *A* is the cross sectional area of the equivalent model which is equal to the sectional area of the skin depth area *A_sk_*.

Considering the distribution of magnetostrictive force is not uniform in both the axial and radial direction, *L_e_* should be adopted the equivalent length in the equivalent model supposing the excitation energy is uniform. Compared with the maximum alternating magnetic field strength obtained in the middle point (*z* = 0) at the surface of the pipe, the distance between two points, where the strengths are 3 dB down at the left and right of the middle point, can be thought as the model length [21]. From Figure 9, the equivalent length can be calculated. *C_e_* is about 1/3.

Substituting Equations from (2) to (5) into (1), the natural frequency is obtained as:

(6) $$f_{n}=\frac{1}{2\pi C_{e}L}\sqrt{\frac{2E}{\rho }}$$

The natural frequency *f_n_* is inversely proportional to the distance *L*, as well as the axial length of the alternating magnetic field.

As analyzed above, the excitation efficiency shall be enhanced when the excitation current frequency is close to the natural frequency of the equivalent model. There is a range of excitation frequency under which the excitation efficiency is enhanced. The center of frequency range is the natural frequency. It is because that the actual excitation current is not a single frequency signal but a bandwidth limited signal with a center frequency. Therefore, if the above special structure is used in the transmitter, as the distance between two copper rings decreases, the natural frequency of the equivalent model increases and the above-mentioned frequency range should be moved to higher frequency domain.

What's more, from Equation (6), it can be seen that the nature frequency *f_n_* of the equivalent model is influenced by the material parameters (*ρ* and *E*) and the equivalent coefficient *C_e_* and the axial length *L*. The equivalent coefficient Ce is determined by the distribution of the excitation magnetic field which is decided by the material parameters (the conductivity and the permeability). Hence, if the material of the pipe keeps unchanged, the relationship between the absolute length *L* and the nature frequency *f_n_* is the same for different pipe sizes.

### 3.3. Limitations of the Equivalent Model

The model present here is an equivalent model of the skin depth area. It is used to calculate the relationship between the axial length of the excitation magnetic field and the nature frequency. However, the nature frequency is only an intermediate parameter. The relationship between the nature frequency and the excitation efficiency can only be analyzed qualitatively.

Therefore, using the equivalent model, the objective of this research, which is the influence of the axial length on the excitation efficiency of the MsS can only be analyzed qualitatively as well. The final quantitative analysis to get the relationship between the axial length of the excitation magnetic field and the excitation efficiency of the MsS must be realized by experiments.

## 4. Experiments and Discussion

### 4.1. The Specimens

**Figure 11.**
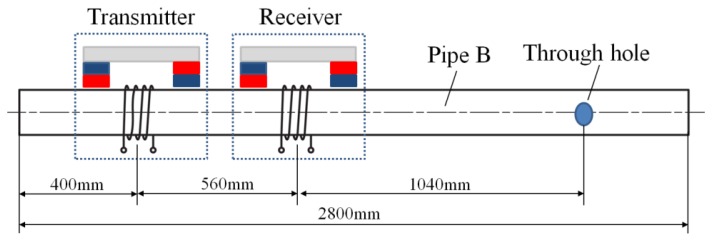
The specimen (pipe B) with a through hole used for defect inspection.

In order to identify the analysis, two pipes, whose materials are both steel 20, are used as the specimens. The Young's modulus is 2.06 × 10^11^ Pa and density is 7,850 kG/m^3^. The outside diameter of the first pipe (pipe A) is 38 mm, wall thickness is 3 mm and length is 2.8 m. The outside diameter of the second pipe (pipe B) is 25 mm, wall thickness is 2.5 mm and length is 2.8 m. Pipe A is an intact pipe. An artificial defect has been made on pipe B, consisting of a through hole whose diameter is 5 mm, as shown in Figure 11. The distance between the defect and the left end of the pipe is 2 m.

**Figure 12.**
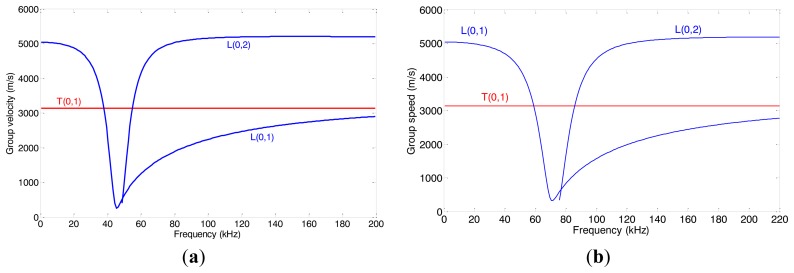
The group velocity dispersion curve of both pipes. (**a**) pipe A. (**b**) pipe B.

**Figure 13.**
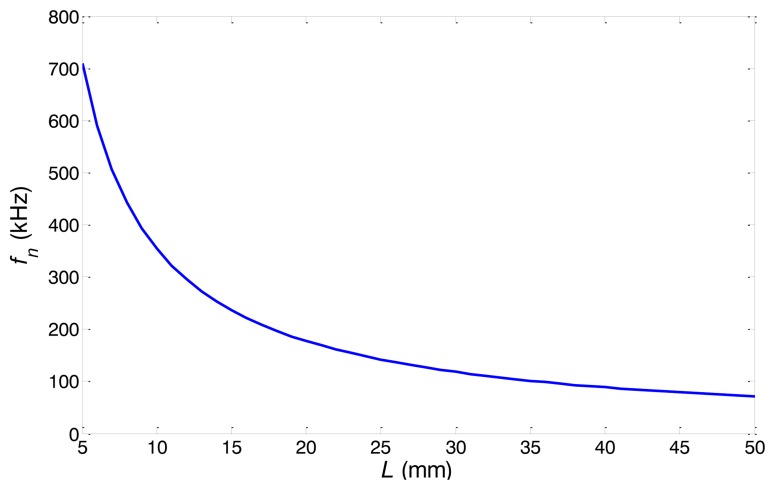
The relationship between *L* and *f_n_*.

The frequency dispersion curves of both pipes are shown in Figure 12. In order to keep the L(0,2) mode non-dispersive, the inspection frequency range of pipe A is set from 80 kHz to 180 kHz and the inspection frequency range of pipe B is set from 120 kHz to 200 kHz. The relationship between natural frequency *f_n_* and the distance *L* is calculated using Equation (6) which is the same for both pipes, as shown in Figure 13. To set the natural frequency in the inspection frequency range, four different distances *L* (20, 25, 30 and 40 mm) are chosen for pipe A. The distances *L* for pipe B are chosen according to the experimental results of pipe A.

### 4.2. Experiment Setup

**Figure 14.**
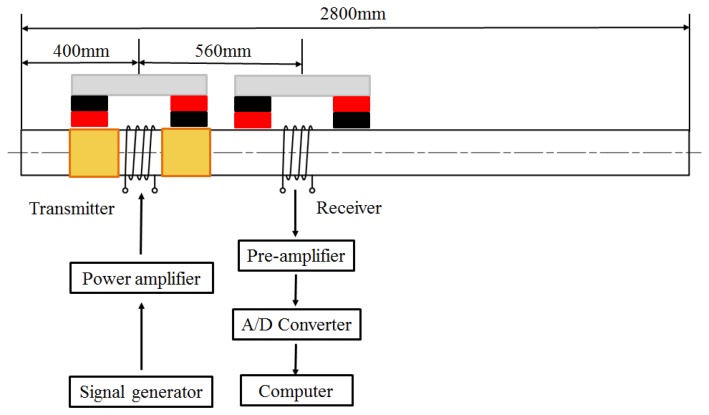
The experimental arrangement diagram.

The experimental set-up is illustrated in Figure 14. The transmitter is placed on the pipe 400 mm away from the left pipe end. The receiver is placed with a distance of 560 mm from it. Each of them contains two permanent magnets for providing a suitable static magnetic field in the pipe. Both the transmitter coil and the receiver coil are 20-turn solenoid coils which consist of copper wire (1 mm outer diameter) wrapped around the pipe. The width is 20 mm and the lift-off is 1 mm. A sine pulse with two circles is generated by a function generator and amplified by a power amplifier.

The amplified sine pulse current is provided to the transmitter coil. The received voltage signal of the receiver coil is amplified by a pre-amplifier, then digitized and sampled by a 16-bits data acquisition card and finally interfaced to a computer.

### 4.3. The Experiment Results

#### 4.3.1. Experiments on Pipe A

**Figure 15.**
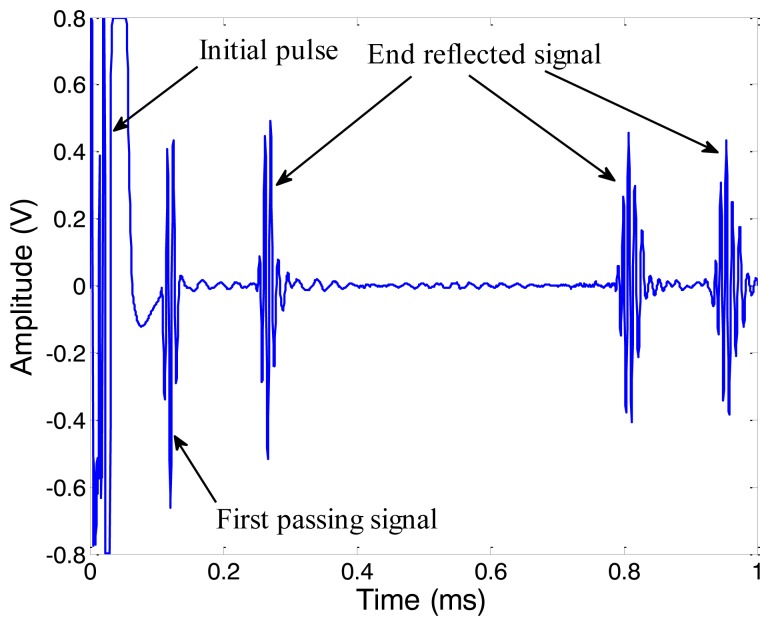
The typical data of the experiments on the intact pipe (pipe A) obtained at 30 A *I_pp_* of excitation current.

**Figure 16.**
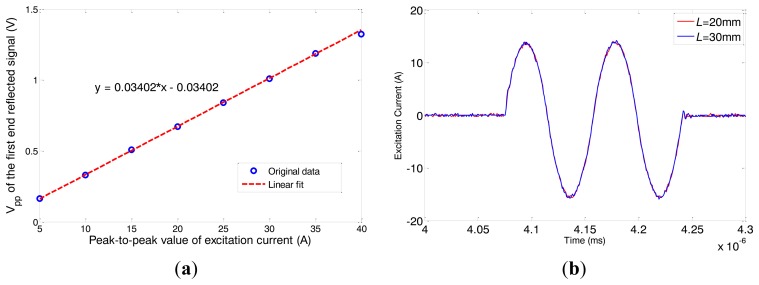
(**a**) The relationship between excitation current and the first reflected signal strength. (**b**) The typical excitation current signals with different *L*.

The amplitude of received signal is in direct proportion to the strength of the alternating magnetic field if the magnetostriction is linear 12. Firstly we change the peak-to-peak amplitude of the excitation current (*I_pp_*) from 5 A to 40 A (in 5 A steps), with a frequency of 120 kHz. The typical data obtained under 30 A *I_pp_* is shown in Figure 15. The first passing signal occurs just after the initial pulse at about 0.106 ms, from which we can calculate that the velocity of wave is about 5,290 m/s, exactly the group speed of L(0,2) mode. We get the peak-to-peak amplitude of the first reflected signal under different excitation currents and the relationship between them is shown as Figure 16a. The *V_pp_* of the first end reflected signal is nearly linear to the *I_pp_* of the excitation current when the *I_pp_* is below 35 A. We choose 30 A as the *I_pp_* of the excitation current so that the magnetostriction is linear in the whole experiment.

To observe the actual effect of the alternating magnetic field axial length variations, experiments on pipe A were done under five different conditions using the conventional structure without the copper ring and the special structure with the two copper rings (*L* = 20, 25, 30 and 40 mm). The excitation frequency is varied from 80 kHz to 180 kHz (step by 10 kHz). The *V_pp_* of excitation voltage signal is set to be constant. The excitation current signals of typical structures are shown in Figure 16b when the applied frequency is 120 kHz. It can be seen that the excitation currents are almost the same for the different structures. In other words, there is little variation in the coil impedance.

**Figure 17.**
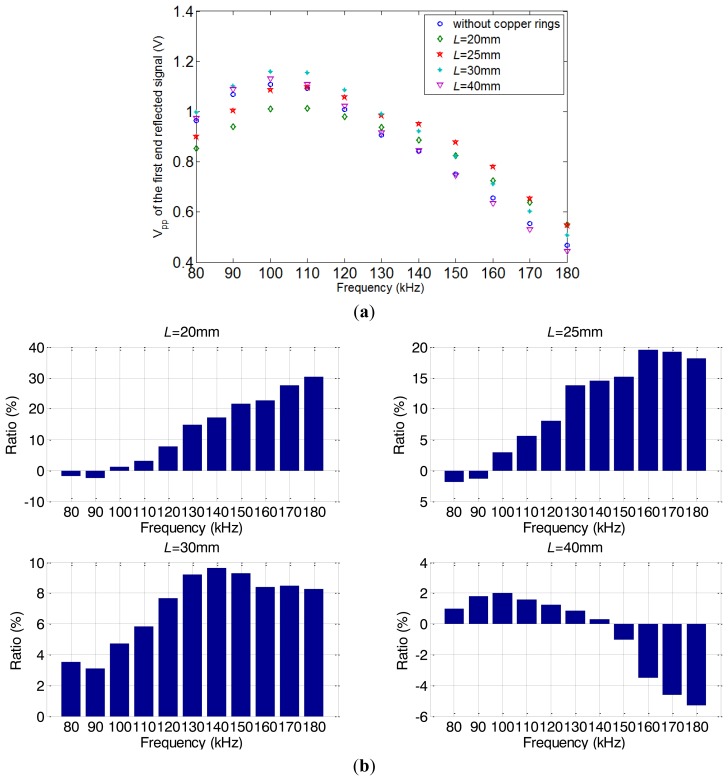
The experiment results of pipe A. (**a**) The peak to peak amplitudes (*V_pp_*) of the first end reflected signals under five conditions. (**b**) The relative enhancement ratios of compensated *V_pp_* with four different *L*, compared with the conventional transmitter.

The relationship between excitation frequency and the peak-to-peak amplitude of the first end reflected signals under different conditions (without copper rings, *L* = 20, 25, 30 and 40 mm) are shown in Figure 17a. Compared with the signal obtained using the conventional transmitter, when the *L* is different, the *V_pp_* of the first reflected signals is enhanced in certain ranges. However, the highest excitation efficiencies are still all obtained in the range from 100 kHz to 110 kHz. This means that these enhancements do not change the general trend of the transmitter's frequency characteristic. For a further analysis, the strengths of alternating magnetic field using the special transmitter are shown in Table 1. In order to compensate the influence of strength, the compensation ratios is obtained compared with the condition *L* = 40 mm. Figure 17b shows the relative enhancement ratios of the *V_pp_* (after multiplying by the compensation ratios) using the special transmitter comparing with the conventional transmitter.

It is obvious that the enhancements of excitation efficiencies are all different under the same excitation frequency. As analyzed above, all these differences are due to the variations of the axial length. For each *L*, the relative enhancement ratio reaches the maximum in a certain frequency *f_L_*, the natural frequency of the equivalent model. Except for *L* = 20 mm, it may because the *f_L_* is larger than 180 kHz. This result agrees well with the theoretical analysis. Table 1 shows the comparison between *f_L_* and natural frequency *f_n_* obtained from Equation (4). The calculated natural frequencies *f_n_* have a relative tolerance around 15%. This may due to the equivalent coefficient *C_e_* should be smaller than 1/3 *L*. The equivalent coefficient *C_e_* can be corrected to 0.2899 for pipe A.

Furthermore, when *L* = 30 mm, the excitation efficiency is enhanced 2%–9% (before compensating for the influence of the strength) in the whole range from 80 kHz to 180 kHz. However, according to the FEM results in Section 2, the alternating magnetic field strength is weaker than the conventional structure. Thus the enhancement effect is only due to the increase of the excitation efficiency caused by adjusting the axial length of the excitation magnetic field.

#### 4.3.2. Experiments on Pipe B

As discussed in Section 3.2, the relationship between the axial length and the excitation efficiency of the MsS should be the same for the pipes with the same material. To verify this point, experiments on pipe B have been done. The dispersion curve of pipe B is shown in Figure 12b. In order to keep the L(0,2) mode non-dispersive, the inspection frequency range is set from 120 kHz to 200 kHz. The experimental set up is the same as in the former experiments.

**Figure 18.**
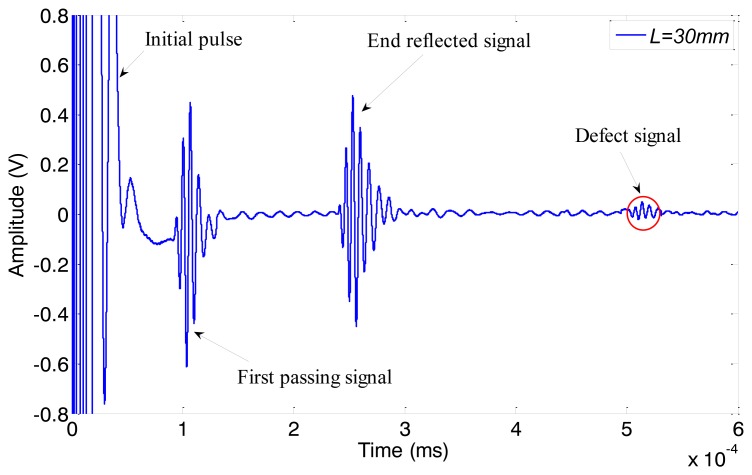
The typical signal obtained on pipe B when *L* = 30 mm.

**Figure 19.**
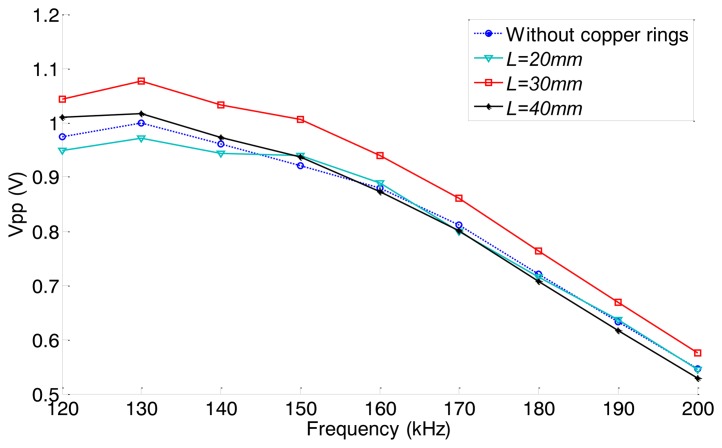
The peak to peak amplitudes (*V_pp_*) of the first end reflected signals under four conditions (without copper rings, *L* = 20 mm, *L* = 30 mm, *L* = 40 mm).

According to the results of the former experiments, the axial length *L* is adjusted from 20 mm to 40 mm (in 10 mm steps). The typical signal obtained when *L* = 30 mm is shown in Figure 18. The relationships between excitation frequency and the peak-to-peak amplitude of the first end reflected signals under four different conditions (without copper rings, *L* = 20 mm, *L* = 30 mm, *L* = 40 mm) are shown in Figure 19. The excitation efficiency of the non-contact MsS has been enhanced 5%–13% in the whole inspection frequency range when the absolute length *L* is 30 mm compared with the excitation efficiency obtained without copper rings. Experiment results show that the optimal absolute lengths *L* for both pipes with the same material are the same, although their sizes are different.

**Figure 20.**
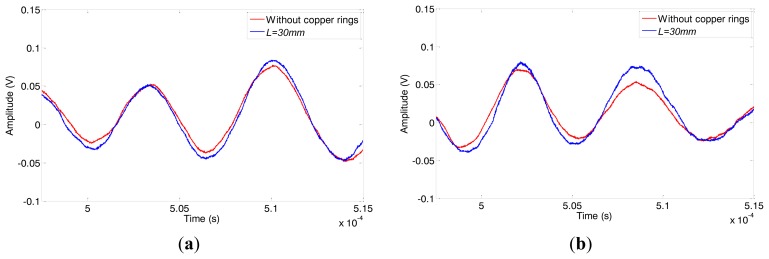
The comparison of defect signals obtained under (**a**) 140 kHz and (**b**) 170 kHz.

The comparison of defect signals obtained under 140 kHz and 170 kHz under two conditions (without copper rings and *L* = 30 mm) are shown in Figure 20. The amplitudes of defect signals are enhanced 4.6% and 9.1%, respectively. The results agree well with the conclusions discussed above.

To sum up, the proposed method of designing a suitable excitation magnetic field range can enhance the excitation efficiency of non-contact MsS used in GWT. The enhancement effect can be obtained under the inspection frequency range of L(0,2) mode. Moreover, the suitable excitation magnetic field range is the same for pipes of the same material but of different sizes.

## 5. Conclusions

This paper provide a method to enhance the excitation efficiency of the non-contact MsS used in GWT in the inspection frequency range of the L(0,2) mode. Unlike other methods of enhancing the excitation efficiency of the non-contact MsS, the method is based on adjusting the axial length of the excitation magnetic field at the expense of weakening the strength of the excitation magnetic field. A special structure is used to adjust the axial length. An equivalent model of the skin depth area is present based on the analysis of the generation progress. The relationship between the axial length of the excitation magnetic field and the natural frequency is calculated using the model. Then the influence of the axial length on the excitation efficiency of the MsS is discussed qualitatively. The excitation efficiency of the non-contact MsS should be enhanced in the frequency range around the nature frequency of the skin depth area. The final influence is analyzed by experiments.

The experimental results fit well with the qualitative analysis. The proposed method of designing a suitable excitation magnetic field range can enhance the excitation efficiency of non-contact MsS used in GWT. The enhancement effect can be obtained in the whole inspection frequency range of L(0,2) mode rather than a single frequency. The method proposed here does not increase the cost and complexity of the inspection system. Moreover, the suitable excitation magnetic field range is the same for the pipes of the same material but different sizes. It means that the optimal axial length obtained on one pipe can be used for the other pipes of the same material. The steps of the proposed method can be summarized as follows:

(1) Obtain the Young's modulus *E* and the density *ρ* of the pipe.
(2) Calculate the dispersion curve of the pipe and choose the inspection frequency range of L(0,2) mode.
(3) Set the natural frequency *f_n_* in the above inspection frequency range and calculate the range of length *L*, which is the distance between the two copper rings, through Equation (6).
(4) Do the experiments according to the ranges of the length *L* and the inspection frequency.
(5) Get the relationship between absolute length *L* and the excitation efficiency of the MsS under different frequencies.
(6) Select the optimal absolute length *L* under which the excitation efficiency of the MsS is enhanced in the whole inspection frequency range.

The method proposed in this paper is based however on a qualitative analysis using the equivalent model and the quantitative analysis based on the experiments. The quantitative relationship between the axial length of the excitation magnetic field and the excitation efficiency of the non-contact MsS used in GWT can only be obtained through experiments. A comprehensive finite element model or theoretical model [22–24], including all the parameters in the inspection progress, is needed for the quantitative analysis. This will be the topic in further research.

## Figures and Tables

**Table 1.** The compensation ratio of *V_pp_* and the comparison of the *f_L_* and natural frequency *f_n_* for different *L*.

**The Value of *L* (mm)**	**Strength of the Alternating Magnetic Field (A/m)**	**Compensation Ratio of *V_pp_***	***f_L_* (kHz)**		***f_n_*(kHz)**		**Relative Tolerance**	
20	6892	1.11		-		172		-	
25	7299	1.05		160		138		13.7%	
30	7499	1.02		140		115		17.8%	
40	7651	1		100		86		14%	
